# Membranous nephropathy with pulmonary cryptococcosis with improved 1-year follow-up results: A case report

**DOI:** 10.1515/med-2021-0234

**Published:** 2021-02-18

**Authors:** Peipei Zhang, Chundan Wang, Jiudan Zhang, Wenjing Zhong, Hong Xia

**Affiliations:** Department of Nephrology, The First Affiliated Hospital of Zhejiang Chinese Medical University, Hangzhou, China; Department of Pathology, The First Affiliated Hospital of Zhejiang Chinese Medical University, Hangzhou, China; Department of Endocrinology, The First Affiliated Hospital of Zhejiang Chinese Medical University, Hangzhou, China; Department of Medical Image, The First Affiliated Hospital of Zhejiang Chinese Medical University, Hangzhou, China

**Keywords:** *Cryptococcus neoformans*, membranous nephropathy, pulmonary cryptococcosis, anti-fungal drugs

## Abstract

Cryptococcosis is frequently found in immunosuppressed patients. It is also a significant opportunistic infection in non-immunocompromised individuals. In this study, we present a rare case of membranous nephropathy (MN) with pulmonary cryptococcosis. A 33-year-old man with MN was referred to our hospital because of dyspnea and weakness for 1 week. Before the above symptoms occurred, the dose of Cyclosporin A was increased again for relapse of MN. Multiple massive or patchy high-density shadows were present on computed tomography of the lung. Initially the patient underwent empirical anti-bacterial therapy, which turned out to be ineffective. As the results of serum cryptococcal latex agglutination tests were positive, the administration of anti-fungal drugs was prescribed. The results of fungal culture and pathologic examination of the lung tissue revealed the findings consistent with *Cryptococcus neoformans*. The patient was successfully treated with voriconazole followed by fluconazole with satisfactory result. Therefore, in patients with chronic kidney disease, lung lesions with poor bactericidal effects of cephalosporins need further examination to make sure whether there is pulmonary cryptococcosis. Early diagnosis and treatment might contribute to good results. It is a problem worthy of consideration that whether immunosuppressive agents need to be discontinued or not during antifungal therapy.

## 1 Introduction

Cryptococcosis is a potentially serious fungal disease associated with significant morbidity and mortality [1]. Clinical presentation of cryptococcosis varies from asymptomatic to life-threatening central nervous system (CNS) involvement. Previous large-scale retrospective analyses have shown that pulmonary cryptococcosis occurs predominantly in patients with HIV. Additional predisposing factors include underlying solid organ transplantation [2], aggressive cancer treatment, use of immunosuppressants or glucocorticoids, connective tissue diseases, or conditions that may damage immune function [3,4,5]. There are fewer cases that have been reported to present with chronic kidney disease with pulmonary cryptococcal infection. In this study, we report a case of membranous nephropathy (MN) with pulmonary cryptococcosis.

## 2 Case report

**Figure 1:**
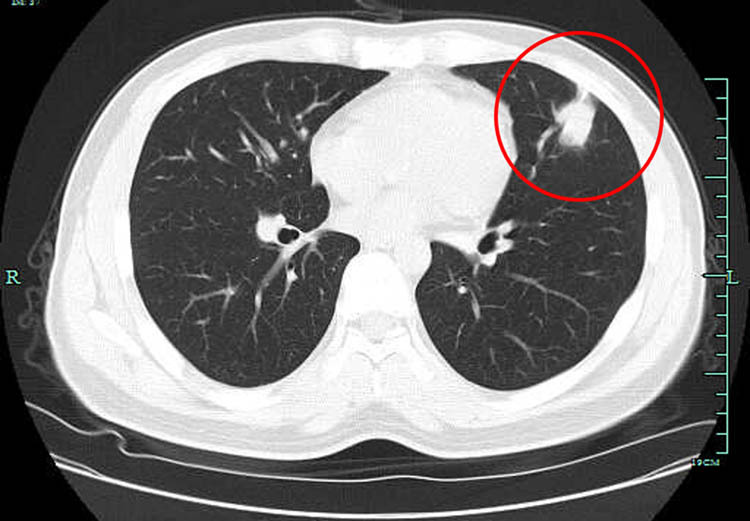
Multiple massive or patchy high-density shadows in the left upper lobe of the lung before any treatment (red circle).

A 33-year-old man with MN was referred to our hospital in February 2018 because of worsening of dyspnea and weakness for 1 week, not accompanied by cough, sputum, or fever. The patient was given a renal biopsy for nephrotic syndrome and diagnosed with as idiopathic MN (IMN) in May 2008 after excluding tumors, hepatitis, and other factors. Treatment with 60 mg of prednisone daily was started after diagnosis of IMN. Because proteinuria continued, treatment was changed to prednisone 30 mg/day and tripterygium glycosides 60 mg/day in August 2008. Six months after prednisone treatment, the patient developed bilateral femoral head necrosis. Then the treatment regimen was adjusted to Cyclosporin A (CsA) 75 mg/12 h. The patient was treated successfully and then controlled with CsA 75 mg/day till 1 year later. Over the next few years, he experienced recurred albuminuria for several times. Even though he remained stable for about 4 years after his most recent discharge, he still required CsA 25 mg/12 h to control recurrence of his disease. Because proteinuria relapsed, treatment with 75 mg of CsA twice daily was increased again in January 2018 and, 1 month later, dyspnea and shortness of breath occurred. The lung computed tomography (CT) scan in local hospital showed nodules involving the left upper lobe (Figure 1). The dyspnea and shortness of breath worsened in the next days, and the patient was admitted to our hospital on February 8, 2018 for further examination.

On admission, laboratory investigations were as follows: hemoglobin, 13.2 g/dL; hematocrit, 38.4%; platelets, 25.1 × 10^4^/µL; massive proteinuria (5.2 g/day) without hematuria; leukocytes, 7,600/µL (72% neutrophils, 22% lymphocytes); total protein, 5.33 g/dL; albumin, 2.75 g/dL; total cholesterol, 0.27 g/dL; blood urea nitrogen (BUN), 31.7 mg/dL; Scr, 0.92 mg/dL; C3, 131 mg/dL; C4, 32 mg/dL; IgG, 794 mg/dL; IgA, 109 mg/dL; IgM, 165 mg/dL; erythrocyte sedimentation rate (ESR) 41 mm/h. C-reactive protein (CRP) and anti-HIV antibody were negative (Table 1).

**Table 1:** Laboratory test results before and after treatment

	February 9, 2018	February 26, 2018	March 5, 2018
WBC (10^9^/L)	7.6	8	8.8
NE (%)	72	59.3	55.3
LY (%)	22	29.4	31.6
HGB (g/dL)	13.2	12.4	12.7
HCT (%)	38.4	35	37.3
PLT (10^9^/L)	251	297	295
CRP (mg/L)	<1	<1	<1
ESR (mm/h)	41	30	6
Anti-HIV1 + 2	Negative	—	—
TP (g/L)	53.3	59	61.7
ALB (g/L)	27.5	31	32.6
CHOL (mmol/L)	7.01	—	—
BUN (mg/dL)	31.7	77.4	84.6
Scr (mg/dL)	0.92	0.67	0.74
C3 (mg/dL)	131	—	—
C4 (mg/dL)	32	—	—
IgG (mg/dL)	794	—	—
IgA (mg/dL)	109	—	—
IgM (mg/dL)	165	—	—
24 h urine protein (g)	5.2	—	0.336
Sputum bacterial culture	Negative	—	—
Blood cryptococcal antigen	—	Positive	Negative

**Figure 2:**
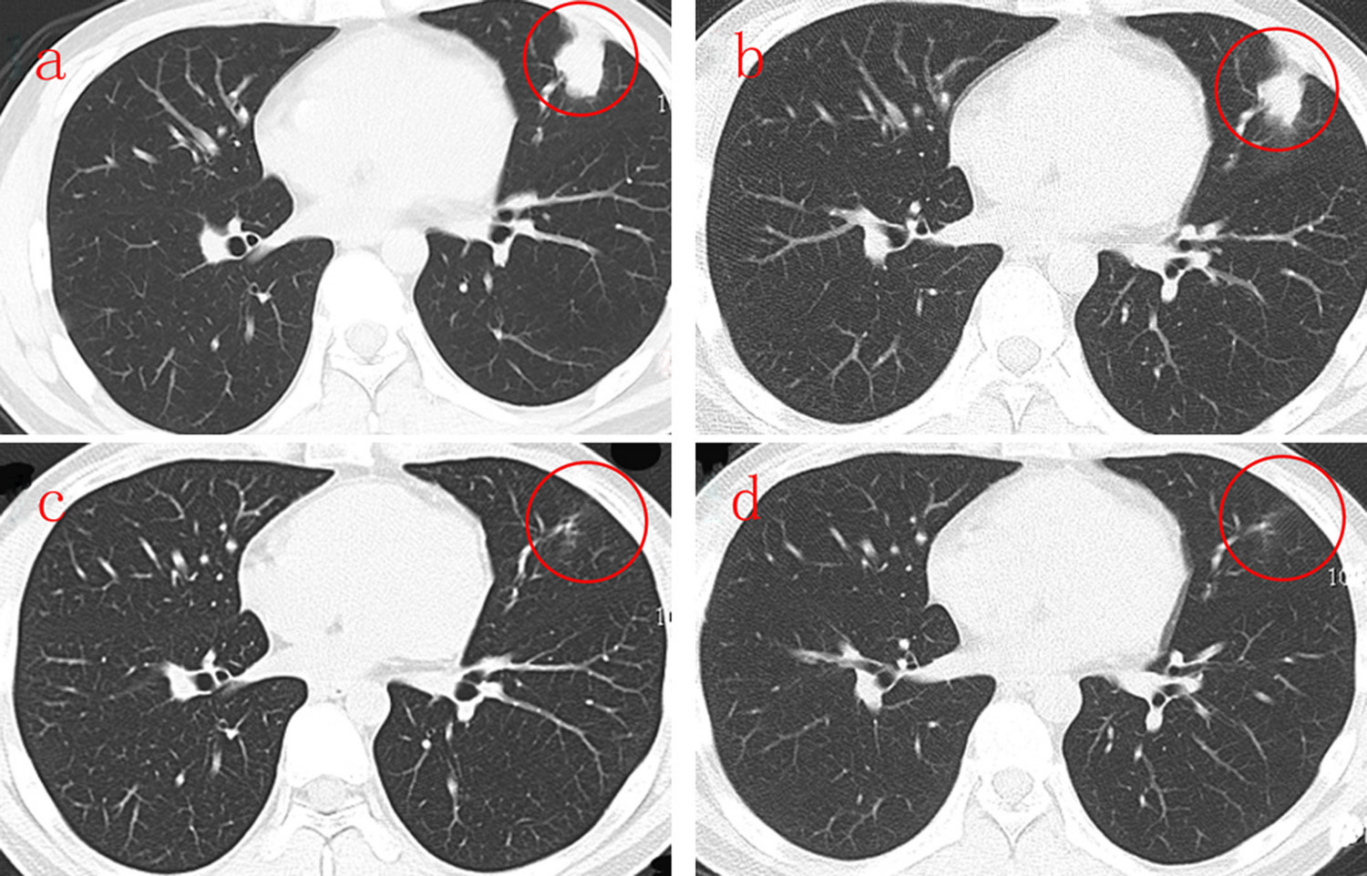
(a) Multiple massive or patchy high-density shadows in the left upper lobe of the lung. (b) The massive or patchy high-density shadows in the left upper lobe of the lung shrink after treatment for 2 weeks. (c) The massive or patchy high-density shadows in the left upper lobe of the lung disappear after treatment for half year. (d) The massive or patchy high-density shadows in the left upper lobe of the lung disappear after treatment for 9 months (red circle).

**Figure 3:**
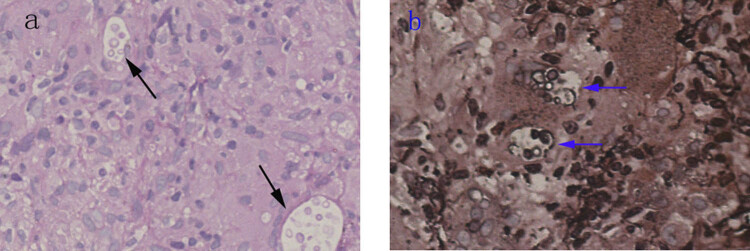
(a) PAS stain of patient’s lung tissue shows many yeast-like cells at 40× magnification (black arrow). (b) PAS-M stain of patient’s lung tissue shows yeast capsules stained in black at 60× magnification (blue arrow).

Treatment with intravenous Piperacillin–Tazobactam was started at a dose of 3.375 g/8 h from February 8 for 1 week. Then chest CT scan was performed and presented no improvement of the nodules involving the left upper lobe (Figure 2a). At this time, serum cryptococcal antigen revealed positive. To confirm the diagnosis, a CT-guided lung biopsy was performed, and fresh lung tissue was submitted for fungal culture. Pathologic examination of the tissue biopsy revealed multiple round shape organisms. The capsules of the observed organisms could be visualized by periodic acid-Schiff (PAS) and periodic acid-silver methenamine (PASM) staining, which is compatible with *Cryptococcus* spp. (Figure 3). The tissue fungal culture showed *Cryptococcus neoformans*, and the chest abnormality was diagnosed as pulmonary cryptococcosis. Furthermore, lumbar puncture and examination of cerebrospinal fluid (CSF) were performed. CSF culture for fungus and cryptococcal antigen were all negative.

Voriconazole was administered intravenously at a dose of 200 mg/12 h from February 15 for 1 week, followed by fluconazole at a dose of 200 mg/day from February 22, 2018 for economical reason. Considering the massive proteinuria at this patient, the administration of oral CsA was maintained at a fixed dose of 75 mg/12 h as before hospitalization. On March 1, a second chest CT showed a decrease in the size of the nodule in the area of left upper lobe (Figure 2b) and serum cryptococcal antigen was negative. On the contrary, interestingly, the amount of proteinuria decreased after the initiation of anti-fungal administration, and subsequently normalized. The patient was discharged on March 5 and he took the oral fluconazole tablets at the same dose for 9 months with regular follow-up. The other two chest CT scans in September and December 2018, respectively, revealed complete remission of the lesion in the left upper lobe (Figure 2c and d). To date, relapse of pulmonary cryptococcosis has not been seen in the ambulatory patient.


**Ethical approval:** The research related to human use has complied with all the relevant national regulations and institutional policies, and is in accordance to the tenets of the Helsinki Declaration and has been approved by the authors’ institutional review board or equivalent committee.
**Informed consent:** Written informed consent was obtained from the patient for publication of this case report and all accompanying images. A copy of the written consent is available for review by the editor of this journal.

## 3 Discussion

Cryptococcosis is an opportunistic infectious disease caused by encapsulated yeasts in the genus *Cryptococcus*. In humans, it is usually caused by the following two subspecies of the *Cryptococcus* spp. family: *C. neoformans* and *C. gattii* [6,7]. *C. neoformans* can cause cryptococcosis in both immunocompetent and immunocompromised patients, whereas *C. gattii* usually infects apparently immunocompetent. Once inhaled into the human host, it colonizes the pulmonary system. The infection can either localize or disseminate through blood to various organs, depending on patients’ immune status. In humans, *C. neoformans* can cause cryptococcal meningitis and wound or cutaneous cryptococcosis except for pulmonary cryptococcosis [8].

In the present case report, the patient was diagnosed as MN and presented with pulmonary cryptococcosis caused by *C. neoformans* without cryptococcal meningitis or cutaneous cryptococcosis. The reports of pulmonary fungal infection in patients with kidney disease are increasing in recent years, for example, kidney transplant patients [9]. The confirmed risk factors cited for fungal infection in kidney transplant patients include demographic (race and older age), medication-associated (immunosuppressive agents), and clinical data (diabetes, urinary tract infection, bacterial pneumonia, bacteremia, and leukopenia/pancytopenia), as well as organism-specific factors [9]. Wang et al. concluded that  immunoglobulin G titer, plasma CsA concentration, serum creatinine level, CD4+/CD8+ ratio, and plasma albumin level were the risk factors of pulmonary infection in primary MN receiving CysA [10]. In recent years, there are some reports of fungal infection in dialysis patients [11] as well as in non-dialyzing uremia [12,13]. Abbott reported that diabetes, female patients, decreasedweight, serum creatinine at initial of dialysis, chronic obstructive lung disease, and AIDS were associated with fungal infection in dialysis patients [11]. Only a few reports have described the relationship between glomerular diseases with or without nephrotic syndrome and fungal infections, e.g., minor glomerular abnormality associated with pulmonary cryptococcosis [14], membranoproliferative glomerulonephritis with Candida endocrinopathy [15], crescentic glomerulonephritis with pulmonary aspergillosis [16], necrotizing glomerulonephritis with pulmonary cryptococcosis [17], and minimal change nephrotic syndrome with cutaneous cryptococcosis published in recent years [18]. In our case, the patient lived in the countryside near mountains, and 6 months before the onset of his chest tightness and shortness of breath, he hunted pheasants many times. His cryptococcosis may be related to this kind of wild bird. Besides, he had been taking oral immunosuppressive agents for more than 10 years, which may cause an immunocompromised host and can be associated with the infectious diseases. Combined with nephrotic syndrome and hypoproteinemia, the malnutrition may also contribute to the patient’s pathological condition. Accordingly, the patient’s definite cause of the development of pulmonary cryptococcosis remains unknown.

What needs to be mentioned is that the correlation between different immunosuppressive agents and cryptococcal infection is distinct. Some studies show that not all immunosuppressive agents would increase the incidence of cryptococcal infection. Mody et al. demonstrated that CsA treatment of mice enhanced survival after inoculation of *Cryptococcus neoformans* by both the intratracheal (IT) and intravenous (IV) routes [19]. Next year he demonstrated that CsA was effective for the treatment of extraneural cryptococcal infection in normal mice [20]. Odom et al. found that growth of the opportunistic fungal pathogen *Cryptococcus neoformans* was sensitive to CsA and FK506 at 37°C but not at 24°C, suggesting that CsA and FK506 inhibit a protein required for *C. neoformans* growth at elevated temperature [21]. These findings identify CsA as a potential antifungal drug. However, CsA was also reported to exacerbate cryptococcal meningitis in both mice and rabbits, most likely because CsA does not effectively penetrate the CNS [21,22]. Complex phenomena require more in-depth research to clarify. Leitheiser et al. assessed the risk factors associated with invasive fungal infections in kidney transplant patients, which suggested that CsA were not associated with increased risk for any patients, neither in Histoplasmosis and Aspergillosis groups nor in Candida, Cryptococcosis, and “Other” mycoses groups [9]. However, Wang and his colleagues reported that the plasma concentration of CsA was associated with pulmonary infections [10].

According to the related article for the management of pulmonary cryptococcosis in asymptomatic non-immunosuppressed patients, localized cryptococcal infection can be treated with fluconazole alone (200–400 mg/day orally) for 6 months [23]. For mild-to-moderate symptoms, administer fluconazole (400 mg/day orally) for 6–12 months. Persistently positive of serum cryptococcal antigen titers are not criteria for continuance of therapy. For severe disease, treatment is similar to disseminated cryptococcosis, as follows: the treatment is divided into induction therapy, consolidation therapy, and maintenance therapy. Induction therapy with amphotericin B deoxycholate (AmBD) 0.5–1.0 mg/kg/day plus flucytosine (100 mg/kg/day) for at least 4 weeks, followed by consolidation therapy with fluconazole (400 mg/day orally) for 8 weeks, and maintenance therapy with fluconazole (200 mg/day) for a total duration of 6–12 months [1]. We treated the patient with fluconazole alone (200 mg/day) for nearly 9 months and saw a rapid clinical response to fluconazole. Interestingly, the decrease in the severity of proteinuria was in parallel with improvement of pulmonary cryptococcosis.

In the case, the MN of this patient was a unique aspect. Proteinuria relapsed 1 month before the cryptococcal infection, and proteinuria remission almost coincided with the lung infection control. Whether some causal relationship exits is not clear. Probably, the relapse of nephrotic syndrome in this case is related to cryptococcal infection. It could not be demonstrated because the dose of CsA did not change in the whole course of antifungal treatment. Kidney biopsy puncture could be the useful method to clarify the causal relationship if cryptococcal antigens are found in kidney tissue. To provide the best treatment, more careful clinical consideration and study are needed when encountering similar problems in the future.
